# Supplementary material for: Processes for creating an interprofessional mental health identity among pre-registration healthcare students: A scoping review protocol
Source: PLoS One. 2025 Nov 13;20(11):e0324568. doi: 10.1371/journal.pone.0324568 (PMC12614503; doi:10.1371/journal.pone.0324568)
Supplement: S1 Appendix — (DOCX) [file pone.0324568.s001.docx]

# S1 Appendix 1 - Medline search strategy

1. interprofession*.tw.

2. interdisciplin*.tw.

3. interoccupation*.tw.

4. interinstitut*.tw.

5. interagen*.tw.

6. exp Interdisciplinary Communication/

7. intersector*.tw.

8. interdepartment*.tw.

9. interorgani?ation$.tw.

10. interprofessional relations/

11. multiprofession*.tw.

12. multidisciplin*.tw.

13. multiinstitution*.tw.

14. multioccupation*.tw.

15. multiagenc*.tw.

16. multisector*.tw.

17. multiorgani?ation*.tw.

18. transprofession*.tw.

19. transdisciplin*.tw.

20. collaborat*.tw.

21. 1 or 2 or 3 or 4 or 5 or 6 or 7 or 8 or 9 or 10 or 11 or 12 or 13 or 14 or 15 or 16 or 17 or 18 or 19 or 20

22. (education* or train* or learn* or teach* or course* or curricul*).tw.

23. Education, Nursing, Associate/ or Education, Medical, Undergraduate/ or Nursing Education Research/ or Education, Graduate/ or Education/ or Education, Pharmacy/ or Education, Pharmacy, Continuing/ or Education, Nursing, Baccalaureate/ or Interprofessional Education/ or Education, Professional/ or Education, Public Health Professional/ or Education, Continuing/ or Education, Medical, Continuing/ or Education, Nursing/ or Education, Nursing, Continuing/ or Education, Nursing, Graduate/ or Education, Medical/ or Education, Dental/ or Education, Pharmacy, Graduate/ or Education, Medical, Graduate/ or Education, Dental, Continuing/

24. student*.tw.

25. 22 or 23 or 24

26. (psychiat* or psycho* or mental* or emot*).tw.

27. exp Mental Health/

28. exp Mental Health Services/

29. 26 or 27 or 28

30. 21 and 25 and 29
