# Supplementary material for: Processes for creating an interprofessional mental health identity among pre-registration healthcare students: A scoping review protocol
Source: PLoS One. 2025 Nov 13;20(11):e0324568. doi: 10.1371/journal.pone.0324568 (PMC12614503; doi:10.1371/journal.pone.0324568)
Supplement: Attachment — Submitted filename: Review Report for PONE.docx [file pone.0324568.s003.docx]

**Review Report for PONE-D-25-20588**

Manuscript Title: Processes for creating an interprofessional mental health identity among pre-registration healthcare students: A scoping review protocol.

Registration: The protocol has been prospectively registered on Open Science Framework Registries (DOI: https://doi.org/10.17605/OSF.IO/TJ9E7).

Overall Recommendation: Revision.

Rationale for Recommendation:

The submission of a scoping review protocol is appropriate for publication in PLOS ONE, as the journal explicitly publishes "Study Protocols" that detail plans for research projects yet to be conducted. This aligns with PLOS ONE's emphasis on scientific validity, robust methodology, and ethical standards. Publishing a protocol enhances research transparency, reduces potential bias, and acknowledges the foundational work before data collection.

However, this protocol requires revision to ensure it fully meets PLOS ONE's expectations for methodological rigor, clarity, and to adequately differentiate its contribution from other review types.

The "Background" section touches upon the concept of interprofessional identity and the gap in understanding how it's formed in the mental health context for pre-registration students. This provides a good foundation for the necessity of a scoping review.

The "Discussion" section briefly reiterates the knowledge gap and the expected outcome of a conceptual model.

**Recommended Revisions:**

Strengthen the Introduction/Background: While the background establishes the need, it would be beneficial to explicitly and concisely define scoping reviews and their purpose, clearly differentiating them from systematic reviews. This will further justify the chosen methodology and highlight its specific contribution.

Elaborate on Data Synthesis: Although narrative synthesis is mentioned, provide more detail on how the charted data will be analyzed and synthesized to achieve the stated objectives, particularly how it will lead to the development of a conceptual model. Clarify the analytical approach to moving from charted data to a proposed model.

Explicitly Address Reporting Guidelines: While the protocol mentions a PRISMA flow chart for the screening process, it would be beneficial to mention that the final scoping review will adhere to relevant reporting guidelines, such as PRISMA-ScR (Preferred Reporting Items for Systematic Reviews and Meta-Analyses extension for Scoping Reviews), to ensure comprehensive and transparent reporting of the eventual findings.

Consider Ethical Statement Nuance: While "N/A" for an ethics statement is permissible, briefly stating why it's not applicable (e.g., "This protocol outlines a review of existing literature and does not involve direct human participants or identifiable data, thus formal ethical approval is not required") could add clarity and preempt potential questions.

By addressing these suggested revisions, the authors can significantly enhance the clarity, rigor, and overall impact of their protocol, demonstrating its valuable contribution to the field in line with PLOS ONE's publication standards.
