# Supplementary material for: Processes for creating an interprofessional mental health identity among pre-registration healthcare students: A scoping review protocol
Source: PLoS One. 2025 Nov 13;20(11):e0324568. doi: 10.1371/journal.pone.0324568 (PMC12614503; doi:10.1371/journal.pone.0324568)
Supplement: Attachment — Submitted filename: Responsetoreviewersv3_protocol_plosone.docx [file pone.0324568.s006.docx]

Mental Health and Addictions Research Group

Department of Health Sciences

University of York

York

United Kingdom

To whom it may concern

We would like to thank you for taking the time to consider our paper entitled ‘Processes for creating an interprofessional mental health identity among pre-registration healthcare students: A scoping review protocol’ for publication in PLOS One. The article provides an overview of a scoping review protocol to map the available literature on processes that have been used to create an interprofessional mental health identity for healthcare students.

We want to thank the reviewers for their time and consideration. We have responded to the comments in the table below, and the changes to the manuscript can be seen in the tracked version. We hope that these changes have improved the quality of the manuscript.

Best wishes,

Claire Carswell

# Response to reviews

| **Reviewer 2** | |
| --- | --- |
| **Comment** | **Response** |
| Unfortunately, there are still many typos throughout the protocol. I suggest having another person read through and flag additional spelling and grammar issues since the journal does not provide copyediting | Thank you for highlighting this. The manuscript has been proof read and also put through Grammarly spell checker to ensure any typos have been flagged and corrected. |
